# Association between eating behavior and quarantine/confinement stressors during the coronavirus disease 2019 outbreak

**DOI:** 10.1186/s40337-020-00317-0

**Published:** 2020-09-01

**Authors:** Chadia Haddad, Maha Zakhour, Maria Bou kheir, Rima Haddad, Myriam Al Hachach, Hala Sacre, Pascale Salameh

**Affiliations:** 1 Research Department, Psychiatric Hospital of the Cross, P.O. Box 60096, Jall-Eddib, Lebanon; 2 INSERM, Univ. Limoges, CH Esquirol, IRD, U1094 Tropical Neuroepidemiology, Institute of Epidemiology and Tropical Neurology, GEIST, Limoges, France; 3 Faculty of Science, Lebanese University, Fanar, Lebanon; 4 Faculty of medicine, Paris Sud University, Rue Gabriel Péri, 94270 Le Kremlin-Bicêtre, Paris, France; 5 Department of Linguistics and Philosophy, Uppsala University, Uppsala, Sweden; 6 Faculty of Engineering, Lebanese university, Roumieh, Lebanon; 7 INSPECT-LB: Institut National de Santé Publique, Epidemiologie Clinique et Toxicologie –Liban, Beirut, Lebanon; 8 Faculty of Pharmacy, Lebanese University, Hadat, Lebanon; 9 Faculty of Medicine, Lebanese University, Hadat, Lebanon

**Keywords:** Quarantine, Confinement, Coronavirus disease, COVID-19, Shape concern, Weight concern, Eating behavior and eating disorder

## Abstract

**Background:**

Quarantine/confinement is an effective measure to face the Coronavirus disease 2019 (COVID-19). Consequently, in response to this stressful situation, people confined to their homes may change their everyday eating behavior. Therefore, the primary objective of this study is to evaluate the association between quarantine/confinement stressors and eating behavior during the COVID-19 outbreak. The secondary objective is to compare the association of quarantine/confinement stressors and diet behavior between two groups of participants, those attending diet clinics and those not (general population).

**Method:**

A cross-sectional web-based online survey carried out between April 3 and 18, 2020, enrolled 407 participants from the Lebanese population. Eating Disorder Examination – Questionnaire (EDE-Q) were used to measure the behavioral features of eating disorders.

**Results:**

More than half of the sample (53.0%) abide by the home quarantine/confinement, 95.4% were living with someone in the quarantine/confinement, and 39.6% continued to work from home. Higher fear of COVID-19 was found in 182 (44.8%) participants, higher boredom in 200 (49.2%) participants, higher anger in 187 (46.3%), and higher anxiety in 197 (48.5%) participants. Higher fear of COVID-19 (Beta = 0.02), higher BMI (Beta = 0.05), and physical activity (Beta = 1.04) were significantly associated with a higher restraint score. Higher anxiety, higher fear of COVID-19, higher BMI, practicing physical exercise, and a higher number of adults living in the quarantine/confinement were significantly associated with higher shape and weight concerns.

**Conclusion:**

Our results showed that the fear of COVID-19 was correlated with more eating restraint, weight, and shape concerns in the whole sample, but more specifically in the dietitian clients group. Public health control measures are needed to reduce the detrimental effects of psychological distress associated with quarantine/confinement on eating behaviors during the COVID-19 outbreak.

## Plain English summary

Under stressful and fearful situations, such as during the Coronavirus disease 2019 (COVID-19), changes in everyday eating behavior might occur. A sample of 407 participants, divided into two groups, one from the general population and the other selected among people attending dietitian clinics, were recruited to study the impact of quarantine and confinement stressors and eating behavior during the COVID-19 outbreak. The quantitative analysis revealed that more than half of the sample abided by home quarantine/confinement, and almost half of them had a higher fear of COVID-19. The latter was associated with higher weight and shape concerns among the total sample, and more specifically, in the dietitian clients group. Public health control measures are needed to define factors of eating disorders during the quarantine/confinement period related to the COVID-19 outbreak and promote healthy habits to lower the risk of psychological distress.

## Background

Quarantine and confinement are the only known effective measures to face the Coronavirus disease 2019 (COVID-19) caused by the novel severe acute respiratory syndrome coronavirus 2 (SARS-CoV2). The first cases of COVID-19 were detected on November 17, in Wuhan, a city in the Hubei province in China, where the outbreak was first identified [1]. The World Health Organization (WHO) declared COVID-19 as a pandemic on March 12, 2020, after the disease spread in several countries, mainly Europe, with more than 20,000 confirmed cases and almost 1000 deaths among Europeans [2]. As a result, a third of the world population adopted the lockdown strategy to face the propagation of the virus and limit the catastrophic effect of its contagious spread, in the absence of an effective vaccine or treatment.

Lebanon, a developing Middle Eastern country, recorded the first COVID-19 case on February 21, 2020. This number raised to 13 on March 1, and one death was reported ten days later. On March 15, the government announced a public health emergency and a national lockdown. By the end of March, the official numbers recorded 446 confirmed cases and 11 deaths and increased to reach a total of 704 cumulative cases and 24 deaths by April 26, 2020 [3]. Furthermore, the risk of psychological distress seemed higher than in other countries, and confinement measures more difficult to endure. Among 15 countries studied in different regions of the world, Lebanon ranked fifth in the prevalence of any mental disorder [4, 5]. This small middle-income country has a long history of civil war and persistent political, social, and economic instability [5]. Recently, a massive economic and political crisis has hit the country, worsened by the economic slowdown due to the spread of the COVID-19 pandemic [6]. Thus, Lebanon entered a double-edged fight against both the disease and an unprecedented financial crisis [6]. Confinement policies became increasingly ineffective as more people feel obliged to return to work to afford their living costs [6].

However, people who respected the sanitary lockdown may have changed their everyday eating behavior due to quarantine/confinement [7]. Indeed, humans are generally sociable, and this period of social isolation may have put them under pressure psychologically, causing some of them to eat more in quantity or frequency as a mechanism to cope with growing fear and anxiety [8]. Stressful and fearful situations are associated with various behavioral responses, with conflicting coping strategies, such as over- or under eating [9]. Some individuals tend to overeat in response to emotional triggers, which leads to more concerns and self-evaluation of body weight or shape [10]. Following bad news about COVID-19 spread, many people may eat more foods without doing any activities, which may lead to weight disturbance [11]. Evidence suggests that the majority of people tend to change their eating behavior when they feel stressed, with about 80% of them altering their caloric intake by either increasing or decreasing their consumption [12]. Also, bored people are likely to eat more than in a controlled state [13]; studies showed that normal weight and overweight people reported eating more when they were lonely or bored [14].

All these factors, namely, social isolation, fear of COVID-19, anxiety, feelings of loneliness, and boredom, have shown to influence eating behavior. People attending diet clinics could be the most affected by eating behavior, weight, and shape concern. Social distancing will not allow them to be followed and controlled by their dietician; instead, they are more at home, with food close hand, and not doing any physical activity. Many of these patients following a specific diet will have rigid and inflexible eating behavior due to the limited range of foods, and the unavailability of some brands recommended by the dietician. Thus, understanding their impact on shape and weight may help predict better outcomes during this critical period. Based on the literature, it seemed reasonable to hypothesize that confinement stressors would be associated with increased weight and shape concerns and that these stressors would be more detected among people who attend a diet clinic than those who do not. Therefore, the primary objective of this study is to evaluate the association between quarantine/confinement stressors and eating behavior during the COVID-19 outbreak. The secondary objective is to compare the association of quarantine/confinement stressors and diet behavior between two groups of participants, those attending diet clinics and those not (general population).

## Methods

### Study design and sampling

A cross-sectional web-based online survey carried out between April 3 and 18, 2020, enrolled 407 participants. Two groups of participants were included in the study: the first consisted of participants selected from the general population; the second included people attending diet clinics for weight loss management, expected to have more weight and eating behaviors related problems. Dieticians were contacted, based on the list retrieved from the Lebanese Academy for Nutrition and Dietetics website, to form this group [15].

For the general population group, the questionnaire was distributed via social media (WhatsApp, Facebook, Instagram), using a snowball technique. For the second group, it was sent by e-mail and WhatsApp to targeted participants selected by the dieticians. The questionnaire required approximately 20 min to complete.

All people above 18 with access to the internet were eligible. The anonymity of the participants was guaranteed during the data collection process (de-identification before data entry and analysis).

### Procedure

The online survey consisted of a link to an internet-based questionnaire on Google forms with closed-ended questions in English and Arabic. Data from completed forms were imported into a Microsoft Excel spreadsheet and analyzed using the SPSS software, version 25.

### Questionnaire

The questionnaire consisted of two parts. The first part assessed the socio-demographic details of the participants (age, gender, marital status, educational level, employment status, region, and the current value of monthly income, divided into four levels: no income, low < 1000 USD, intermediate 1000–2000 USD, and high income > 2000 USD), and their Body Mass Index (BMI).

The BMI was calculated by dividing self-reported (due to the confinement) weight (in Kg) by height (in m^2^). Participants were then classified into four categories, according to their BMI: underweight (< 18.5 kg/m2), normal (18.5–24.9 kg/m2), overweight (25.0–29.9 kg/m2), and obese (≥30.0 kg/m2) [16].

The second part of the questionnaire consisted of a set of nine questions related to stressors of quarantine and confinement, in addition to various scales:

#### Quarantine and confinement stressors

Under this category, a set of nine questions defining the stressors of quarantine/confinement were retrieved from previous articles [17, 18]. The questions were about “Closed and prolonged coexistence with the family member”, “Financial difficulty due to quarantine/confinement”, “Difficulty buying the desired foods and products”, “Constant sense of insecurity for oneself and loved ones”, “Physical exercise practice during quarantine/confinement”, and “Lack of physical contact with friends”. Additionally, questions regarding the length of quarantine/confinement in days and the numbers of adults and children living in the same house during quarantine/confinement were also asked.

#### Current fear of COVID-19

Ten questions selected from previous studies were used to assess the current fear of COVID-19 in people [19–22]. Examples of the asked questions: “Thinking about COVID-19 makes me feel anxious”, “I feel tense when I think about the threat of COVID-19”, and “I feel quite anxious about the possibility of another outbreak of COVID-19”. All items were measured on a 5-point Likert scale, from 1 (not at all) to 5 (extremely). The total score ranged from 10 to 50. High scores indicated a greater fear of COVID-19 infection. In this study, the Cronbach’s alpha value was 0.917.

By the time our data collection was completed, a study validating a fear of the COVID-19 scale was published [23], and thus could not be used in this paper.

#### Short boredom proneness scale (SBPS)

The SBPS is a self-report questionnaire consisting of eight items rated on a 7-point Likert scale ranging from 1 (strongly disagree) to 7 (strongly agree) [24]. The total score ranged from 8 to 56. Higher scores indicated a greater tendency to boredom [24]. Permission to use the scale for the current article was obtained from the author of the questionnaire (Pr. *James Danckert).* In this study, the Cronbach’s alpha value was 0.912.

#### Lebanese anxiety scale (LAS)

This 10-item self-report scale, recently developed and validated in Lebanon, was created to screen for anxiety [25]. Seven of the items are graded on a 5-point Likert scale (0 = Not present to 4 = very severe) and the remaining three, on 4-point Likert scale (1 = almost never to 4 = almost always) [25]. The total score was obtained by summing all the responses, with higher scores indicating higher anxiety [25]. In this study, the Cronbach’s alpha value was 0.884.

#### Anger subscale of the Buss-Perry scale

The Buss-Perry Scale is a 29-item questionnaire composed of four factors that measure physical and verbal aggression, anger, and hostility [26]. In this study, the anger subscale (8 items) was used and was graded on a 5-point Likert scale from 1 (extremely uncharacteristic of me) to 5 (extremely characteristic of me) [26]. The total score was calculated by summing all the responses, with higher scores indicating a higher anger score. In this study, the Cronbach’s alpha value was 0.865.

#### Eating disorder examination questionnaire (EDE-Q)

The Eating Disorder Examination-Questionnaire (EDE-Q) is a 28-item self-reported tool measuring the range and severity of behavioral features of eating disorders [27, 28]. It is rated using four subscales and a global score. The four subscales are restraint, eating concern, shape concern, and weight concern, and reflect the severity of eating disorders. All items are scored on a 7-point rating scale (0–6), higher scores indicating greater levels of symptomatology [28]. In this study, the Cronbach’s alpha values of the four subscales were as follows: restraint subscale (Cronbach’s alpha = 0.835), eating concern (Cronbach’s alpha = 0.745), shape concern (Cronbach’s alpha = 0.902), and weight concern (Cronbach’s alpha = 0.824).

### Translation procedure

A forward and backward translation was conducted for all the scales except for the LAS-10 already available in Arabic. One translator was in charge of translating the scales from English to Arabic, and a second one performed the back translation. Discrepancies between the original English version and the translated one were resolved by consensus.

### Statistical analysis

Data were analyzed using Statistical Package for Social Sciences (SPSS software version 25). A descriptive analysis was done using the counts and percentages for categorical variables and mean and standard deviation for continuous measures. Pearson correlation analyses were used for continuous variables, and Student t-test and ANOVA F tests for categorical variables with two or more levels, to assess the association of variables with the continuous scales.

As we have a four subscales of behavioral eating disorders, four stepwise linear regressions were conducted, taking the EDE restraint subscale, EDE-eating concern subscale, EDE-shape concern subscale, and EDE-weight concern subscale as the dependent variables. The stepwise method was used to simultaneously remove the weakest correlated variables and come up with a model that best explains the distribution. All variables that showed a *p* < 0.1 in the bivariate analysis were included in the model to eliminate potential confounding factors as much as possible. All variables that showed a *p* < 0.1 in the bivariate analysis were included in the model to eliminate potential confounding factors as much as possible [29]. Afterward, the same analysis was conducted on the stratified data (general population and dietician clients groups), using the same set of dependent and independent variables. A value of *p* < 0.05 was considered significant. The reliability of the scales was assessed using Cronbach’s alpha.

## Results

### Sample description

The results showed that the mean age of the participants was 30.59 ± 10.10 years (Mode: 26.00; range: 55), with 51.3% females. The mean BMI of the participants during the quarantine/confinement was 25.08 ± 4.44 Kg/m2. Only 10 participants were underweight (2.5%), 218 (53.8%) had normal weight, 124 (30.8%) were overweight, and 52 (12.9%) were obese. Also, the dietitian clients group had significantly higher BMI and age as compared to the general population group (Table 1).

**Table 1:** Sociodemographic characteristics of the participants

	Total sample (***n*** = 407)	General population group (***N*** = 228 (56.3%))	Dietitian clients group (***N*** = 177 (43.7%))
	Frequency (%)	Frequency (%)	Frequency (%)
**Gender**			
Male	198 (48.7%)	93 (40.8%)	105 (59.7%)
Female	209 (51.3%)	135 (59.2%)	71 (40.3%)
*p-value*		< 0.001	
**Marital status**			
Single	305 (75.0%)	180 (79.0%)	123 (69.4%)
Married	102 (25.0%)	48 (21.0%)	54 (30.6%)
*p-value*		0.030	
**Education level**			
University level	370 (90.9%)	214 (93.9%)	153 (86.8%)
Secondary level and below	37 (9.1%)	14 (6.1%)	23 (13.2%)
*p-value*		0.017	
	**Mean ± SD**		
**BMI (Kg/m**^**2**^**)**	25.08 ± 4.44	22.00 ± 1.91	29.05 ± 3.55
*p-value*		< 0.001	
**Age**	30.59 ± 10.10	28.33 ± 7.48	33.52 ± 12.11
*p-value*		< 0.001	

### Quarantine and confinement stressors

Table 2 describes the quarantine/confinement situation and stressors among the participants. In the absence of cut-off values for fear of the COVID-19 scale, boredom scale, anger subscale, and anxiety scale, the median was considered as a cut-off point. Higher fear of COVID-19 was found in 182 (44.8%) participants, higher boredom in 200 (49.2%) participants, higher anger in 187 (46.3%), and higher anxiety in 197 (48.5%) participants.

**Table 2:** Description of the quarantine/confinement situation and stressors among the participants (N=407)

	Frequency	Percentage
**Quarantine/confinement stressors**		
**Closed and prolonged coexistence with the family**		
Yes	327	80.4%
No	80	19.6%
**Financial difficulty due to the quarantine/confinement**		
Yes	140	34.5%
No	267	65.5%
**Difficulty buying the desired food and products**		
Yes	114	28.0%
No	293	72.0%
**Lack of physical contact with friends**		
Most of the time	189	46.5%
Some of the time	22	5.4%
Rarely	58	14.4%
Never	137	33.7%
**Constant sense of insecurity for themselves and loved ones**		
Yes	174	42.8%
No	233	57.2%
**Physical exercise practice during quarantine/confinement**		
Yes	240	58.9%
No	167	41.1%
	**Mean**	**SD**
**Fear of COVID-19 scale**	28.49	9.19
**Short Boredom Proneness scale**	24.12	11.79
**Length of quarantine/confinement in days**	26.05	10.69
**Number of adults living in the quarantine/confinement**	3.21	1.30
**Number of children living in the quarantine/confinement**	0.54	0.96

### Bivariate analysis: correlates of eating behaviors

In the total sample, a higher restraint mean score was significantly associated with the practice of physical activity during quarantine/confinement, and greater fear of COVID-19 was significantly but weakly associated with restraint score. A significantly higher eating, shape and weight concerns mean score were found in dietitian clients’ group participants, those who have financial problems, those who had a constant sense of insecurity, and those who practiced physical activity during the quarantine/confinement. Also, greater fear of COVID-19, boredom, anxiety, and anger, were significantly associated with higher eating, shape, and weight concerns scores. It is noteworthy that the association between abiding by the home quarantine and EDE was not significant (Table 3).

**Table 3:** Bivariate analysis taking the eating behaviors as the dependent variables in the total sample

	EDE restraint subscale		EDE eating concern subscale		EDE shape concern subscale		EDE weight concern subscale	
	M ± SD	*p*- value	M ± SD	*p-*value	M ± SD	*p-*value	M ± SD	*p-*value
**Groups of participants**								
Participants from the general population group	1.13 ± 1.42	0.051	0.83 ± 1.12	**< 0.001**	1.45 ± 1.46	**< 0.001**	1.13 ± 1.38	**< 0.001**
Dietitian clients group	1.45 ± 1.74		1.30 ± 1.28		2.09 ± 1.78		1.78 ± 1.62	
**Abide to the home quarantine**								
Yes	1.34 ± 1.63	0.335	1.07 ± 1.22	0.555	1.86 ± 1.70	0.121	1.50 ± 1.58	0.298
No	1.19 ± 1.50		1.00 ± 1.21		1.60 ± 1.56		1.34 ± 1.47	
**Closed and prolonged coexistence with the family**								
Yes	1.32 ± 1.59	0.636	1.16 ± 1.31	0.565	1.90 ± 1.75	0.610	1.60 ± 1.70	0.891
No	1.42 ± 1.62		1.26 ± 1.36		2.01 ± 1.78		1.63 ± 1.53	
**Financial difficulty due to quarantine/confinement**								
Yes	1.54 ± 1.70	0.076	1.44 ± 1.44	**0.005**	2.25 ± 1.98	**0.009**	1.90 ± 1.86	**0.015**
No	1.23 ± 1.52		1.04 ± 1.23		1.74 ± 1.60		1.45 ± 1.54	
**Difficulty buying desired food**								
Yes	1.47 ± 1.84	0.309	1.46 ± 1.55	**0.010**	2.21 ± 1.94	**0.042**	1.84 ± 1.86	0.080
No	1.28 ± 1.48		1.06 ± 1.19		1.80 ± 1.66		1.51 ± 1.58	
**Lack of physical contact with friends**								
Most of the time	1.55 ± 1.67	0.058	1.28 ± 1.48	0.058	2.21 ± 1.88	0.001*	1.88 ± 1.82	< 0.001*
Some of the time	1.07 ± 1.42		1.26 ± 1.28		2.24 ± 1.53		1.95 ± 1.44	
Rarely	1.21 ± 1.57		1.36 ± 1.26		1.86 ± 1.57		1.59 ± 1.54	
Never	1.11 ± 1.48		0.92 ± 1.02		1.43 ± 1.56		1.12 ± 1.40	
**Constant sense of insecurity for themselves and loved ones**								
Yes	1.49 ± 1.67	0.101	1.58 ± 1.60	**< 0.001**	2.39 ± 1.99	**< 0.001**	2.01 ± 1.93	**< 0.001**
No	1.23 ± 1.53		0.88 ± 0.97		1.58 ± 1.47		1.32 ± 1.39	
**Physical exercise practice during quarantine/confinement**								
Yes	1.68 ± 1.70	**< 0.001**	1.31 ± 1.40	**0.011**	2.06 ± 1.76	**0.045**	1.76 ± 1.68	**0.021**
No	0.84 ± 1.26		0.99 ± 1.16		1.71 ± 1.73		1.38 ± 1.63	
	**Correlation coefficient**	*p-***value**	**Correlation coefficient**	*p-***value**	**Correlation coefficient**	*p-***value**	**Correlation coefficient**	*p-***value**
**Length of quarantine/confinement in days**	0.073	0.142	0.080	0.106	0.109	**0.027**	0.113	**0.023**
**Number of adults living in the quarantine/confinement**	0.069	0.164	0.086	0.083	0.108	**0.028**	0.106	**0.032**
**Number of children living in the quarantine/confinement**	0.013	0.789	0.014	0.773	−0.029	0.556	−0.043	0.387
**Fear of COVID-19 scale**	0.120	**0.015**	0.237	**< 0.001**	0.246	**< 0.001**	0.192	**< 0.001**
**Short Boredom Proneness scale**	0.035	0.484	0.254	**< 0.001**	0.253	**< 0.001**	0.250	**< 0.001**
**Anxiety scale**	0.064	0.194	0.357	**< 0.001**	0.332	**< 0.001**	0.304	**< 0.001**
**Anger scale**	0.044	0.373	0.191	**< 0.001**	0.202	**< 0.001**	0.186	**< 0.001**

*Bonferroni post-hoc analysis: Association between lack of physical contact with friends and shape concern subscale: Most of the time vs. some of the time *p* = 1.000, most of the time vs. rarely *p* = 1.000, most of the time vs. never *p* < 0.001, some of the time vs. rarely *p* = 1.000, some of the time vs. never *p* = 0.231, rarely vs. never *p* = 0.773

Association between lack of physical contact with friends and weight concern subscale: Most of the time vs. some of the time *p* = 1.000, most of the time vs. rarely *p* = 1.000, most of the time vs. never *p* < 0.001, some of the time vs. rarely *p* = 1.000, some of the time vs. never *p* = 0.152, rarely vs. never *p* = 0.475.

*p*-value marked in bold are significant (Less than 0.05)

### Multivariable analysis

The results of a first linear regression, taking the restraint scale as the dependent variable, showed that the association was highly significant between a higher restraint score and a greater fear of COVID-19 (Beta = 0.02), higher BMI (Beta = 0.05), and physical activity (Beta = 1.04) (Table 4, Model 1). A second linear regression, taking the eating concern scale as the dependent variable, showed that the association was highly significant between a higher eating concern score and the female gender (Beta = 0.52), higher anxiety (Beta = 0.04), higher BMI (Beta = 0.06), a constant sense of insecurity (Beta = 0.41), and physical activity (Beta = 0.43) (Table 4, Model 2).

**Table 4:** Multivariable analysis in the total sample

Variable	Unstandardized Beta	Standardized Beta	***P***	95% Confidence Interval	
**Model 1: Linear regression variable taking the ‘EDE-Restraint subscale’** **as the** **dependent variable** **and** **the sociodemographic, quarantine/confinement stressors, anger and anxiety** **as the** **independent variables.**					
Physical exercise during quarantine/confinement	1.04	0.32	< 0.001	0.74	1.35
Fear of COVID-19 scale	0.02	0.16	0.001	0.01	0.04
BMI (kg/m^2^)	0.05	0.15	0.002	0.02	0.09
Variables entered in the models: Age, gender, marital status, education level, BMI, fear of COVID-19 scale, short boredom proneness scale, anxiety scale, anger scale, financial difficulty due to the quarantine/confinement and physical exercise during quarantine/confinement.					
**Model 2: Linear regression variable taking the ‘EDE- Eating Concern subscale’** **as the** **dependent variable** **and** **the sociodemographic, quarantine/confinement stressors, anger and anxiety** **as** **the independent variables.**					
Anxiety	0.04	0.28	< 0.001	0.03	0.06
Gender (male^a^ vs. female)	0.52	0.21	< 0.001	0.30	0.74
BMI (kg/m^2^)	0.06	0.25	< 0.001	0.04	0.09
Physical exercise during quarantine/confinement	0.43	0.17	< 0.001	0.20	0.65
Constant sense of insecurity for oneself and loved ones	0.41	0.16	0.001	0.18	0.65
Variables entered in the models: Age, gender, marital status, education level, BMI, fear of COVID-19 scale, short boredom proneness scale, anxiety scale, anger scale, constant sense of insecurity for themselves and loved ones, financial difficulty due to the quarantine/confinement and physical exercise during quarantine/confinement.					
**Model 3: Linear regression variable taking the ‘EDE- Shape Concern subscale’** **as the** **dependent variable** **and** **the sociodemographic, quarantine/confinement stressors, anger and anxiety** **as the** **independent variables.**					
Anxiety	0.05	0.23	< 0.001	0.03	0.07
BMI (kg/m^2^)	0.14	0.39	< 0.001	0.11	0.18
Gender (male^a^ vs. female)	0.63	0.19	< 0.001	0.35	0.91
Fear of COVID-19 scale	0.03	0.20	< 0.001	0.02	0.05
Age	−0.02	− 0.16	0.001	− 0.04	− 0.01
Physical exercise during quarantine/confinement	0.50	0.15	0.001	0.21	0.79
Presence of physical contact with friends	−0.46	− 0.13	0.002	− 0.76	− 0.16
Number of adults living in the quarantine/confinement	0.13	0.10	0.019	0.02	0.23
University education level	−0.55	− 0.09	0.046	−1.08	− 0.01
Variables entered in the models: Age, gender, marital status, education level, BMI, length of quarantine/confinement in days, number of adults living in the quarantine/confinement, fear of COVID-19 scale, short boredom proneness scale, anxiety scale, anger scale, constant sense of insecurity for themselves and loved ones, financial difficulty due to the quarantine/confinement, difficulty buying the desired food and products, presence of physical contact with friends and physical exercise during quarantine/confinement.					
**Model 4: Linear regression variable taking the ‘EDE- Weight Concern subscale’** **as the** **dependent variable** **and** **the sociodemographic quarantine/confinement stressors, anger and anxiety** **as the** **independent variables.**					
Anxiety	0.03	0.19	< 0.001	0.01	0.05
BMI (Kg/m^2^)	0.14	0.41	< 0.001	0.11	0.17
Gender (male^a^ vs. female)	0.63	0.20	< 0.001	0.37	0.89
Physical exercise during quarantine/confinement	0.61	0.19	< 0.001	0.35	0.88
Short Boredom Proneness scale	0.02	0.15	0.002	0.008	0.03
Number of adults living in the quarantine/confinement	0.17	0.15	< 0.001	0.07	0.27
Presence of physical contact with friends	−0.46	− 0.14	0.001	− 0.73	− 0.19
Fear of COVID-19 scale	0.02	0.12	0.008	0.005	0.03

Variables entered in the models: Age, gender, marital status, education level, BMI, length of quarantine/confinement in days, number of adults living in the quarantine/confinement, fear of COVID-19 scale, short boredom proneness scale, anxiety scale, anger scale, constant sense of insecurity for themselves and loved ones, financial difficulty due to the quarantine/confinement, difficulty buying the desired food and products, presence of physical contact with friends and physical exercise during quarantine/confinement.

^a^Reference group

When taking the shape and weight concern scales as the dependent variable, the results showed that higher shape and weight concern scores were significantly associated with the female gender, higher anxiety, greater fear of COVID-19, a higher number of adults living together in the quarantine/confinement, higher BMI, and physical activity. Furthermore, physical contact with friends was significantly associated with lower weight and shape concern scores (Table 4, Model 3 and Model 4).

### Stratification over the two group of participants

Tables 5 and 6 present the results of the stratification analysis performed over the two groups of participants, the general population group and the dietitian clients group. Physical contact with friends was significantly associated with lower weight and shape concern scores in the group of the general population. Higher fear of COVID-19 was significantly associated with higher eating, shape, and weight concern scores in the dietitian clients group.

**Table 5:** Multivariable analysis in the general population group

	Unstandardized Beta	95% CI		*p-*value
**Model 1: Linear regression variable taking the ‘EDE-Restraint subscale’** **as the** **dependent variable** **and** **the sociodemographic, quarantine/confinement stressors, anger and anxiety** **as the** **independent variables.**				
Physical exercise during quarantine/confinement	0.736	0.354	1.118	< 0.001
Gender (Male* vs. female)	0.421	0.013	0.829	0.043
Fear of COVID-19 scale	.006	−.018	.030	.615
Short Boredom Proneness scale	.005	−.016	.026	.637
**Model 2: Linear regression variable taking the ‘EDE- Shape Concern subscale’** **as the** **dependent variable** **and** **the sociodemographic, quarantine/confinement stressors, anger and anxiety** **as the** **independent variables.**				
Anxiety scale	0.057	0.034	0.081	< 0.001
Presence of physical contact with friends	− 0.863	−1.254	− 0.472	< 0.001
Number of adults living in the quarantine/confinement	0.115	−0.037	0.266	0.137
Fear of COVID-19 scale	0.006	−0.018	0.029	0.648
Short Boredom Proneness scale	0.013	−0.007	0.033	0.215
Gender (Male* vs. female)	0.558	0.162	0.955	0.006
Education level (university vs. secondary and lower*)	−0.817	−1.628	− 0.006	0.048
Physical exercise during quarantine/confinement	0.382	−0.005	0.769	0.053
BMI (Kg/m^2^)	0.084	−0.017	0.185	0.101
**Model 3: Linear regression variable taking the ‘EDE- Weight Concern subscale’** **as the** **dependent variable** **and** **the sociodemographic quarantine/confinement stressors, anger and anxiety** **as the** **independent variables.**				
Presence of physical contact with friends	−0.716	−1.094	−0.338	< 0.001
Gender (Male* vs. female)	0.609	0.232	0.986	0.002
Physical exercise during quarantine/confinement	0.435	0.085	0.786	0.015
Fear of COVID-19 scale	−0.009	−0.031	0.014	0.454
Short Boredom Proneness scale	0.021	0.002	0.040	0.031
Number of adults living in the quarantine/confinement	0.112	−0.032	0.256	0.126
BMI (Kg/m^2^)	0.115	0.019	0.211	0.019
**Model 4: Linear regression variable taking the ‘EDE- Eating Concern subscale’** **as the** **dependent variable** **and** **the sociodemographic, quarantine/confinement stressors, anger and anxiety** **as** **the independent variables.**				
Anxiety scale	0.035	0.011	0.059	0.005
Gender (Male* vs. female)	0.694	0.387	1.002	< 0.001
Constant sense of insecurity for oneself and loved ones	0.211	−0.114	0.536	0.202
Physical exercise during quarantine/confinement	0.208	− 0.093	0.509	0.174
Fear of COVID-19 scale	−0.003	−0.021	0.016	0.760
BMI (Kg/m^2^)	0.087	0.007	0.166	0.032

*Reference group

**Table 6:** Multivariable analysis in the dietitian clients group

	Unstandardized Beta	95% CI		*p-*value
**Model 1: Linear regression variable taking the ‘EDE-Restraint subscale’** **as the** **dependent variable** **and** **the sociodemographic, quarantine/confinement stressors, anger and anxiety** **as the** **independent variables.**				
Physical exercise during quarantine/confinement	1.394	0.903	1.886	< 0.001
Gender (Male* vs. female)	0.210	0.373	−0.254	0.674
Fear of COVID-19 scale	0.062	0.036	0.087	< 0.001
Short Boredom Proneness scale	−0.038	− 0.062	− 0.015	0.001
**Model 2: Linear regression variable taking the ‘EDE- Shape Concern subscale’** **as the** **dependent variable** **and** **the sociodemographic, quarantine/confinement stressors, anger and anxiety** **as the** **independent variables.**				
Anxiety scale	0.025	−0.008	0.058	0.136
Presence of physical contact with friends	0.165	−0.295	0.625	0.479
Number of adults living in the quarantine/confinement	0.265	0.102	0.427	0.002
Fear of COVID-19 scale	0.068	0.044	0.092	< 0.001
Short Boredom Proneness scale	0.037	0.017	0.058	< 0.001
Gender (Male* vs. female)	0.569	0.122	1.015	0.013
Education level (university vs. secondary and lower*)	−0.123	− 0.935	0.689	0.765
Physical exercise during quarantine/confinement	0.681	0.228	1.135	0.003
BMI (Kg/m^2^)	0.097	0.031	0.162	0.004
**Model 3: Linear regression variable taking the ‘EDE- Weight Concern subscale’** **as the** **dependent variable** **and** **the sociodemographic quarantine/confinement stressors, anger and anxiety** **as the** **independent variables.**				
Presence of physical contact with friends	0.018	−0.405	0.440	0.934
Gender (Male* vs. female)	0.525	0.102	0.949	0.015
Physical exercise during quarantine/confinement	0.853	0.436	1.270	< 0.001
Fear of COVID-19 scale	0.051	0.026	0.076	< 0.001
Short Boredom Proneness scale	0.027	0.006	0.047	0.013
Number of adults living in the quarantine/confinement	0.274	0.120	0.428	0.001
BMI (Kg/m^2^)	0.118	0.058	0.178	< 0.001
**Model 4: Linear regression variable taking the ‘EDE- Eating Concern subscale’** **as the** **dependent variable** **and** **the sociodemographic, quarantine/confinement stressors, anger and anxiety** **as** **the independent variables.**				
Anxiety scale	0.026	0.001	0.051	0.042
Gender (Male* vs. female)	0.272	−0.069	0.613	0.117
Constant sense of insecurity for oneself and loved ones	0.579	0.191	0.967	0.004
Physical exercise during quarantine/confinement	0.767	0.419	1.114	< 0.001
Fear of COVID-19 scale	0.033	0.013	0.054	0.002
BMI (Kg/m^2^)	0.012	−0.037	0.061	0.641

Higher anxiety was significantly associated with a higher eating concern score in both groups.

## Discussion

To our knowledge, this is the first study to examine the effect of quarantine/confinement stressors due to COVID-19 on behavioral eating disorders among 407 Lebanese participants from all the Lebanese regions. Our results showed that 44.8% of participants had a higher fear of COVID-19, 48.5% had anxiety, and more than half (53%) of the sample were abiding by home quarantine/confinement. A recent study in Wuhan (510 participants) and Shanghai (501 participants) found a moderate to severe anxiety related to the COVID-19 disease [30]. Another research conducted among 1210 participants from 194 cities in China revealed moderate to severe anxiety symptoms in 28.8%, while 8.1% had moderate to severe stress during the first phase of the COVID-19 outbreak, and most of the respondents abided by home quarantine/confinement (84.7%) [31]. Fear and anxiety during the worldwide pandemic, where cities and even entire countries were locked down, might be overwhelming and stressful for people and cause strong and high distress emotions.

In times of uncertainty, people are most vulnerable to different groups of mental disorders that may constitute comorbid disorders [32]. People with high trait anxiety, poor coping strategies, and excessive worrying and fear might develop a major depressive episode that forms a general neurotic syndrome [32]. Previous findings revealed that a population characterized by mixed anxiety and depressive symptoms has a significantly worse long-term outcome than patients without this syndrome [33]. Moreover, studies showed that emotional instability, hypertension, and anxious perfectionism were related to restrained and eating behaviors [34, 35]. Also, people with neuroticism traits having a higher vulnerability when coping with stressful events are at higher risk of eating disorders [35].

Greater fear of COVID-19 was significantly associated with higher eating restraint, consistent with results from previous studies showing that dietary restriction is linked to lower psychological health and higher anxiety [36–39]. The stressful situation imposed by the COVID-19 outbreak and the subsequent quarantine affect the emotional status, resulting in loss of control that might influence eating behaviors [40]. Our results showed that anxiety and higher fear of COVID-19 were associated with higher body shape and weight concerns, in agreement with previous findings showing that anxiety and fear co-occur with eating disorders [41–44]. A person with feelings of intense distress might experience severe disturbances in eating behavior, such an extremely reduced food intake or extreme overeating, which consequently could increase body weight and shape concerns. These results were unexpected, as previous studies revealed that the lockdown and the inability of people to do any physical activity resulted in overeating and drinking, weight gain, and obesity [11, 45]. Indeed, stress and anxiety affect body weight through biological behavioral and psychological mechanisms. Stress can lead to the consumption of a higher quantity of food and reduced physical activity [46, 47]. A recent study showed that during the COVID-19 quarantine, only 22% of the population gained weight, while those who maintained or lost weight were more likely to practice restraint eating [7].

Weight and shape concerns increased with the number of individuals in the quarantine/confinement. A higher number of people living together often drives up the demand for food, typically contributing to disrupted eating patterns, which in turn affects the nutritional status. Physical contact with friends was significantly associated with lower weight concerns. These findings are in agreement with a study showing that higher feelings of loneliness are associated with high weight and shape concern [48]. However, it is noteworthy that connection with peers can have either positive or negative influences on body image, weight, and shape status [49]. Some studies have shown a positive correlation between the connection with peers and weight concerns [50–52].

When looking at the association between quarantine/confinement stressors and eating behaviors among the dietitian clients group and the general population group, the results revealed that higher fear of COVID-19 score and higher boredom were associated with higher disturbed eating behavior in the dietitian clients group. Indeed these hard times could be even more challenging for those trying to manage their weight [18]. Many people find it difficult to control their weight as they tend to fall back on comfort food to help them cope with the stress of COVID-19 lockdown and social isolation [53]. During times when people are most emotionally vulnerable, they tend to lose their ability to control their eating resulting in excessive self-evaluation and worrying about weight gain and weight management issues [54]. Studies are warranted to clarify the difference between dietitian clients and the general population regarding quarantine/confinement stressors and weight and shape concerns.

Finally, media and anecdotal reports suggest that a large percentage of populations are eating better, whether overeating or undereating, now that they have extra time to prepare food and do home cooking, despite being stressed about money, job security, and infection rates. Further studies are needed to explore this aspect.

### Limitations

Although our results are consistent with those of previous research, our study has several limitations. Using a cross-sectional questionnaire-based design does not allow to confirm that merely the fear of COVID-19 caused more restraint eating, weight, and shape concerns; a longitudinal study would better assess the association of the quarantine/confinement on eating disorders. Furthermore, the sample may not be representative of the entire population of quarantined/confined people since the actual number of respondents is relatively low and not heterogeneous. Also, the results could not be generalized to the whole population since the majority of the respondents were well-educated with computer literacy and internet access, which suggests that less-educated people and those unable to access the internet were not assessed.

An information bias could exist since the information was self-reported by the participants; it is not sure whether they were accurate and noted exactly the gain or loss even of a few grams. Furthermore, self-selection bias may have occurred as people with any eating disorder were more motivated to enroll than other participants. The instrument used to assess the current fear of COVID-19 was derived from several surveys and is not yet validated in the Lebanese context. Eating behaviors were not assessed, nor the data and information about eating behaviors, such as the number of meals/ snacks per day, calories consumed, and stances of unplanned eating. This study did not include a matched control group of persons who were not quarantined/confined, which would have allowed the assessment of possible eating disorders in the community at large as an effect of the COVID-19. Residual confounding bias is also possible since there could be factors related to eating behaviors that were not measured in this study. Additionally, further details about participants were not assessed in this study, such as the number of people who stopped going to the gym, the eating status of participants at the time of the study, and prior to the pandemic.

## Conclusion

Although quarantine/confinement is essential to curb the spread of the disease, it generates different negative psychological impacts like fear of infection, anxiety, anger, and boredom. Our results showed that the fear of COVID-19 was correlated with more eating restraint, weight, and shape concerns in the whole sample, but more specifically in the dietitian clients group. Public health control measures are needed to reduce the detrimental effects of psychological distress associated with quarantine/confinement on eating behaviors during the COVID-19 outbreak. Additional support is recommended to people at increased risk for adverse psychological and social consequences of quarantine/confinement.

## Data Availability

Data can be made available under reasonable request form the corresponding author.

## Abbreviations

COVID-19: Coronavirus disease 2019
EDE-Q: Eating Disorder Examination – Questionnaire
BMI: Body Mass Index
SARS: Severe acute respiratory syndrome
SARS-CoV2: Severe acute respiratory syndrome coronavirus 2
WHO: World Health Organization
USD: United States dollar
SBPS: Short Boredom Proneness Scale
LAS: Lebanese Anxiety Scale
SPSS: Statistical Package for Social Sciences
